# The Role of Arthroereisis in Improving Sports Performance, Foot Aesthetics and Quality of Life in Children and Adolescents with Flexible Flatfoot

**DOI:** 10.3390/children9070973

**Published:** 2022-06-29

**Authors:** Alexandru Herdea, Adrian-Gabriel Neculai, Alexandru Ulici

**Affiliations:** 1 11th Department of Pediatric Orthopedics, “Carol Davila” University of Medicine and Pharmacy, 050474 Bucharest, Romania; alexherdea@yahoo.com; 2 Pediatric Orthopedics Department, Grigore Alexandrescu Children’s Emergency Hospital, 011743 Bucharest, Romania; adrianneculai@yahoo.com

**Keywords:** flexible flatfoot, pediatric orthopedics, arthroereisis, subtalar implant

## Abstract

Flexible flatfoot represents one of the most common deformities of the lower limb, affecting children and adolescents. Aesthetic aspect, abnormal gait, pain and fatigue are by far the most important symptoms which determine parents to bring their children to the orthopedist. We set out to conduct a prospective study, case-controlled, including patients with symptomatic flexible flatfeet operated on by arthroereisis surgery and comparing them to a normal feet group of children age- and sex-matched (control group). Minimum follow-up time was 2 years. In total, 33 patients with bilateral arthroereisis were included and 36 patients formed the control group (12.12 +/− 1.85 years vs. 11.81 ± 2.40 years, *p* = 0.54). Quality of life improved postoperatively (*p* = 0.18) and was not different from the control group. Median running time improved postoperatively by 2.25 s (*p* < 0.0001) and got closer to the median running time from the control group (22.30 s compared to 20.94 s, *p* = 0.01). All radiological angles improved (*p* < 0.0001), but quality of life improvement was correlated with talonavicular coverage angle and Meary angle measurements. Flatfoot in children and adolescents may be a condition in which the quality of life and sports performance are decreased, compared to healthy children. Arthroereisis is a minimally invasive surgical procedure with a short recovery time and a short period before resumption of sport activities, which can be useful in certain types of flexible flatfoot due to its effectiveness on symptom reduction.

## 1. Introduction

Flexible flatfoot or flexible pes planovalgus represents one of the most common deformities of the lower limb, affecting both children and adults, consisting of a collapsed medial arch, abduction of the forefoot, medial rotation and plantar flexion of the talus [1]. The condition dates back to Ancient Egypt, with one of Tutankhamen’s mummy’s feet having this disorder [2]. Being the support of the whole body’s weight, the anatomy of the foot consists of a complex network of bones, muscles and ligaments. The architecture of the foot is based on three arches: longitudinal medial, longitudinal lateral and transverse; the first mentioned is the most involved in weight pressure absorption, and its anomaly genuinely characterizes flatfoot [3,4]. All children present with it due to the thickness of the plantar fat pad and joint laxity, but, by the age of five, the emergence of the medial arch begins to correct [5]. The lack of a definitory aspect for a certain diagnosis of flatfeet has made the determination of a real incidence of this defect a real challenge [6].

Flatfoot can be present in three different forms: flexible flatfoot (FFF), flexible flatfoot with short tendo-Achilles (FFF-STA) and rigid flatfoot; the first form is also the most common and the least cause of disability [6].

Paraclinical diagnosis can be confirmed through certain angles visible on radiographical images. The angle between the talus and first metatarsal (Meary), the talo-calcaneal angle (Kite’s angle) in both the AP (anteroposterior) and lateral view, the calcaneal pitch and the talonavicular coverage angle tend to be over their normal ranges in cases of patients with flatfoot [7,8].

The range of motion (ROM) is a test used in order to detect if the patient has a flexible or rigid pes planus, while conditions such as prominent fat pad, edema mimicking flatfoot or neoplasms can be excluded by clinical or imaging evaluation [9]. The Silfverskiöld test may be performed in the case of uncertainty regarding the length of Achille’s tendon, so as to take into consideration or not an intraoperative elongation [10].

Being a condition that affects the foot geometry, a gait analysis is recommended in order to stage the gait anomaly [11]. Depending on the affected structure or function, there are some techniques used to characterize motion health.

The kinematics of the gait involves a digital reconstruction of the subject’s body, requires the use of a 3D motion analysis, and enables the joint angle, velocity and acceleration to be calculated [12].

Even though it provides important data about the gait, the cause of the abnormal gait may not be shown through the kinematics, but through the kinetics which include energy, forces and power generated by individual movements, useful for a better understanding of runners’ gait cycle [12,13].

Because of the continuous progress of technology, gait can nowadays be tested with video methods. Video gait analysis (VGA) is used to record the stride, running gait and movement patterns, parameters that may come in handy during diagnosis or treatment plan establishment [14].

In terms of epidemiology, a slightly higher rate of flatfoot cases was found in boys, but the most important aspect to take into consideration was BMI (body mass index) which showed that for obese patients, as well as for the younger aged patients, flatfoot was more common [15]. Wearing shoes at an early age may jeopardize the longitudinal arch development and, as a result, flatfoot rate; thus, we can affirm that pes planus is more common in urban areas [16].

There are many reasons why parents bring their children with flatfoot to the orthopedic clinics, including aesthetic aspect and abnormal gait; however, by far the most important symptoms are pain and fatigue [17]. The quality of the patient’s life is decreased by the incapacity to perform in sport and through society exclusion; in some countries, individuals with flatfoot cannot be recruited to the army [18].

Flexible flatfoot treatment has included many variations over the years [6]. Conservative treatments such as orthoses, insoles or orthopedic footwear are still used, but the results are modest or uncertain [19]. According to some authors, foot orthoses prescription should be reconsidered for flatfooted patients [20,21,22]. Others mentioned that they may not be recommended due to the lack of evidence of structural improvements for pediatric flexible flatfoot; insoles seem to have a beneficial role for patients under 13, but additional studies may be required in order to confirm their therapeutic effect, and orthopedic footwear did not correct the deficient arch development [23,24,25]. Surgical procedures for flatfoot are continuously updated and some are still used nowadays, such as lateral column calcaneal lengthening described by Evans and modified by Mosca, Grice’s extraarticular arthrodesis and pseudoarthrodesis, better known as arthroereisis [26,27,28,29,30]. Arthroereisis has experienced multiple changes since its introduction, being initially based on the insertion of a bone block inside the sinus tarsi which restricts excessive subtalar joint eversion [28,29,30]. Despite the fact that it is not yet standardized, subtalar arthroereisis’ advantages are the simplicity of procedure, the short-term recovery, effectiveness and the low risk rate [31].

Regarding the numerous pediatric patients diagnosed with flexible flatfoot, the goal of our study is to evaluate if the arthroereisis surgery may eliminate the painful gait and improve the physical condition and the aesthetical aspect for all the patients that experience disfunction in any of these three directions.

## 2. Materials and Methods

### 2.1. Study Design

**Figure 1:**
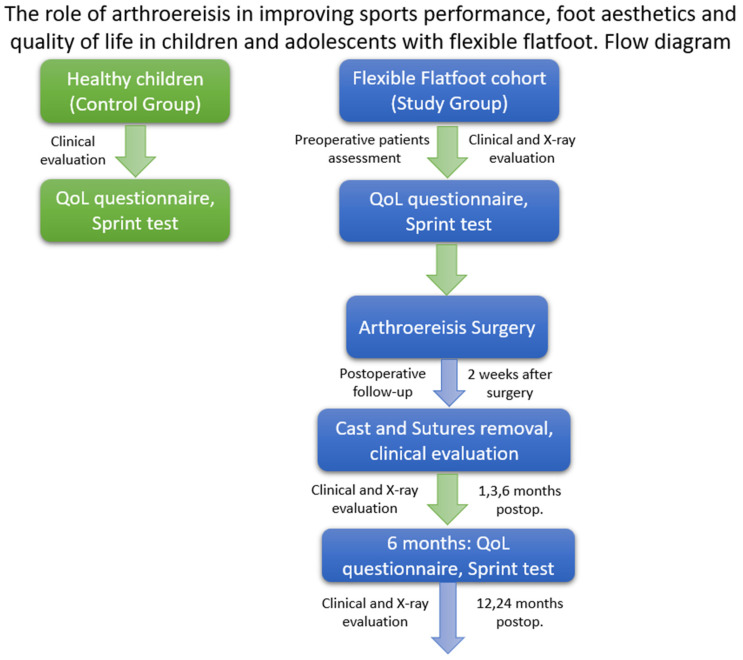
Flow diagram describing the conditions of each stage. **Left** diagram represents the control group with healthy children composed of school children, within the same age range as the study group. **Right** diagram represents the study group composed of the flexible flatfoot cohort.

The study took place in the Pediatric Orthopedics Department of “Grigore Alexandrescu” Children’s Emergency Clinical Hospital, Bucharest, Romania, located in an urban area, between 2018 and 2020. The ethics committee of “Grigore Alexandrescu” Children’s Emergency Clinical Hospital of Bucharest approved this study on 10 January 2018. The identification number of the survey is 10. Informed consent was obtained from the parents of all the participants. A flow diagram is described in Figure 1.

### 2.2. Participants

We set out to conduct a prospective study, case-controlled, that began in 2018 to include patients with symptomatic flexible flatfoot operated on by arthroereisis surgery, and compare them to a normal feet group of age- and sex-matched children.

The working protocol for the patients in the study group included the following aspects: patient history, clinical and radiological exam, a life quality staging questionnaire and a physical exam.

In the patient’s history, the patient and the parents were asked about the occurrence of pain and fatigue in the legs, if they can do sports as much as their colleagues, if there is a threshold after which they begin to get tired and if there is any aesthetic deficit.

### 2.3. Study Procedure

Clinical examination evaluated the foot and ankle ROM, presence/absence of the longitudinal arch, forefoot abduction, calcanean valgus, the Hubscher maneuver (or Jack’s test), presence/absence of hallux valgus, tip-toe walking and walking gait abnormalities [32,33].

**Figure 2:**
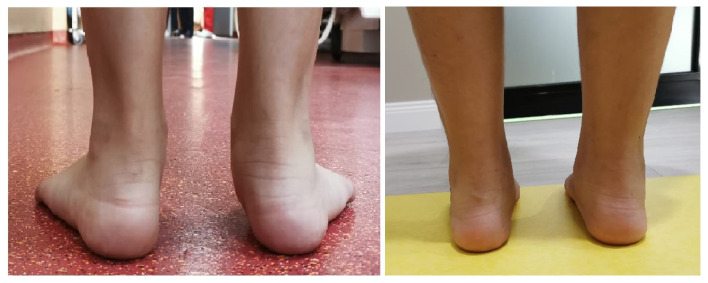
Clinical appearance of the flatfoot seen from behind: Preoperative (**Left**) and postoperative (**Right**). The image shows a flat-footed patient operated on by arthroereisis using a titanium implant without lengthening the Achilles tendon. From our collection of pre- and postoperative photos.

The Achilles tendon was assessed by the Silfverskiöld test in order to determine if surgical lengthening is required or stretching exercises only. Photos and videos of the foot and the walking gait were taken before and 6 months after the surgery, and were electronically stored, as seen in Figure 2. BMI was measured before surgery and at 6 months post-surgery.

Radiological examination consisted of standing anteroposterior and lateral view x-rays that followed the calcaneal pitch, Meary’s angle, Kite’s angle and talonavicular coverage angle. Radiographies were made before surgery, at the end of the surgery and 1, 3, 6, 12 and 24 months, respectively, after the intervention. Digital radiology was used, and measurements were done in a digital environment using the Biotronics3D-3dnet medical.

Quality of life was assessed using AOFAS (The American Orthopaedic Foot and Ankle Society) Ankle-Hindfoot Scale, a survey composed out of a series of questions grouped in 3 categories that followed pain, function and alignment, with a maximum score of 100 points [34]. Parents aided the patients with the completion of the questionnaire. The questionnaires were completed before surgery and 1-year post-surgery.

Physical and sports examination consisted of 3 laps of 100 m sprints, from which the fastest was chosen. Patients warmed up before running laps, and all safety precautions were taken to ensure that no patient was injured. The run was performed on a sports field, with measured asphalt indicating the 100 m stretch. Timing was conducted in seconds and milliseconds.

Inclusion criteria for the study group included patients diagnosed with flexible flatfoot (clinical and radiological exam), patient history, having symptoms of pain and fatigue, starting at the age of nine, with a follow-up period of at least two years after surgery, that were going to have surgery by arthroereisis, with informed consent.

Exclusion criteria for the study group included rigid flatfoot, patients younger than nine years of age, pain due to other syndromes such as Sever or Iselin disease, lack of patient history, clinical or radiological exam, lack of informed consent, and follow-up of less than 2 years.

The control group consisted of school children within the same age range as the study group. Our control group consisted of recreational athletes only, with none performing any sport at a professional level. Children were measured, weighed (BMI) and consulted to rule out a diagnosis of flatfeet, other foot malformations or other associated health problems. They then followed the same running protocol to record the time obtained in the 100-m race. Quality of life was assessed using the AOFAS Ankle-Hindfoot Scale.

### 2.4. Surgical Procedure

**Figure 3:**
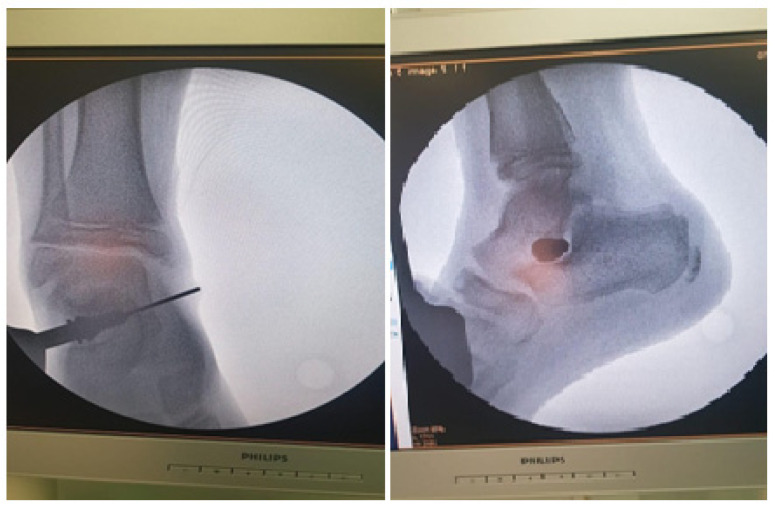
Radiological image made with the image amplifier in the operating room which confirms the final position of the implant. On the **left**, the anteroposterior image; on the **right**, the lateral image. The image includes a pin guide and a titanium implant.

Arthroereisis was performed using a titanium implant which was inserted in the sinus tarsi. The choice of implant was done upon availability at the hospital. Surgery was done by a single vertical incision of 2 cm on the lateral side of the foot in the sinus tarsi area. A template was used inside the sinus tarsi to determine the size of the implant needed. X-ray guidance was used in all the maneuvers. Final size and position of the titanium implant was confirmed by X-ray, and determined by ankle and foot ROM, as seen in Figure 3.

If ankle dorsiflexion was below −10° degrees, Hoke procedure was for Achilles tendon elongation. For ankle dorsiflexion between −10° and 0° degrees, stretching exercises during the physiotherapy session were recommended after surgery in the recovery stage.

**Figure 4:**
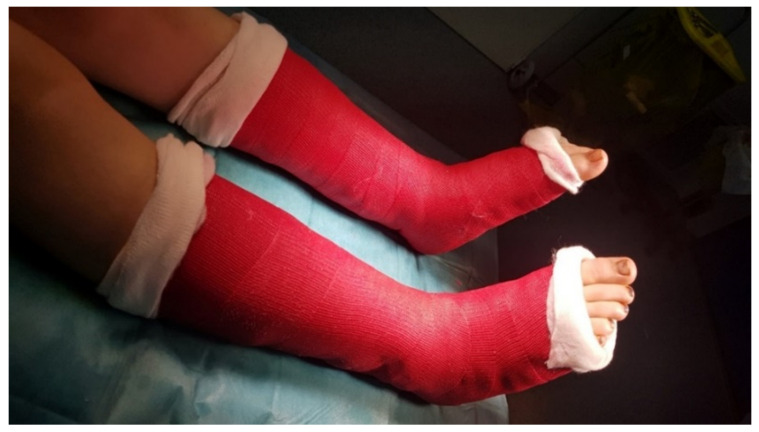
Following the arthroereisis surgery, to relieve postoperative pain, a walking plaster made of resin was applied for two weeks. Two weeks later, the cast and wires were removed, and the normal gait was resumed.

The postoperative protocol followed a walking cast, with patients being encouraged to walk from the second day after the surgery, as seen in Figure 4. After 2 weeks, the cast and wires were removed from the wound.

**Figure 5:**
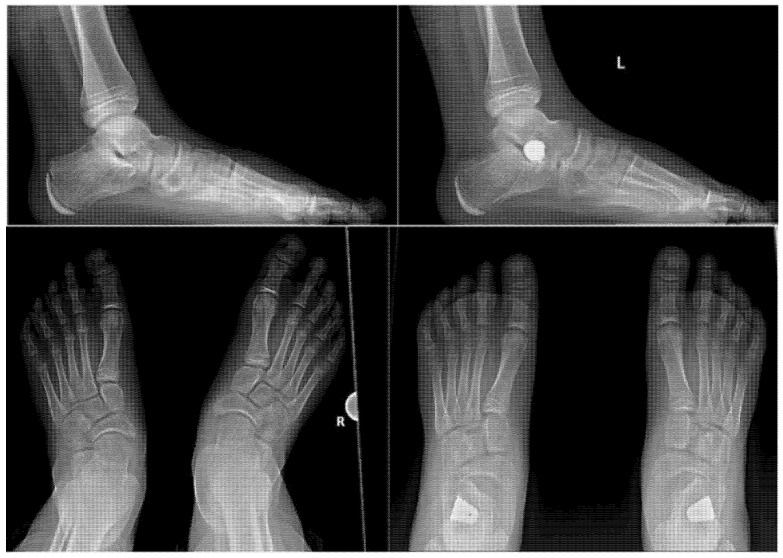
Comparative radiological images taken before surgery (**left**) and one month after surgery (**right**) with significant correction of Meary angle (in the lateral view) and talonavicular coverage angle (in the anteroposterior view).

At 1 month, X-rays were performed in the standing lateral and anteroposterior views, as seen in Figure 5. Pain and mobility were assessed. If pain was present or walking gait was still abnormal, patients underwent a couple of sessions of physiotherapy. Patients were allowed to return to sports 2–3 months post-surgery. Follow-up was conducted at 2 weeks post-surgery, and at 1, 3, 6, 12 and 24 months.

### 2.5. Statistical Analysis

Statistical analysis was performed with GraphPad 9 Prism. Data distribution was tested with the D’Agostino and Pearson test. The *t*-test was used to compare the means if the data had a Gaussian distribution, and the Wilcoxon test was used to compare the medians. The Mann–Whitney test was used to compare BMI medians [35,36,37,38]. We set the significance level at 5% (0.05). Standard deviation (S.D.), minimum (Min.), maximum (Max.), mean and median values were calculated and a confidence interval (CI) of 95% was used. The statistical power of the study was assessed.

The acquired and statistically analyzed data comprised the following variables: age, BMI, QoL in study group between preoperative and postoperative stages, QoL between postoperative study and the control group, median running time between preoperative and postoperative values for the study group, median running time between postoperative study and the controls, radiological angle values between preoperative and postoperative stages, radiological measurement values and QoL improvements.

## 3. Results

The present study includes the study group of 33 patients with bilateral arthroereisis (19 boys and 14 girls) and the control group of 36 patients (20 boys, 16 girls). None of the patients received surgical elongation of the Achilles tendon, with patients having an ankle between −5° and −10° degrees.

The average age of the patients in the study group did not differ statistically significantly from the average age of the patients in the control group (11.93 ± 1.85 years vs. 11.72 ± 1.56 years, *p* = 0.54), as seen in Table 1.

In terms of BMI medians, there is a statistically significant difference in favor of the study group obtained in the Mann–Whitney test (19.07 vs. 16.85 kg/m^2^, *p* < 0.0001).

The quality of life assessment, using the AOFAS Ankle-Hindfoot Scale, is much higher postoperatively than preoperatively, with the median value of the difference being 62 points (*p* < 0.0001), as seen in Table 1.

Postoperatively, the quality of life does not differ statistically significantly from that of the control group where healthy children are included (*p* = 0.18), as seen in Table 1.

The median running time of patients in the preoperative study group was statistically significantly decreased by 2.25 s compared to the median running time in the postoperative period (*p* < 0.0001), as shown in Table 2. Comparing operated children to normal children, we can see that operated children have a longer running time compared to the control group (22.30 s compared to 20.94 s, *p* = 0.01).

Looking at the radiological angles measured preoperatively and postoperatively, we observed statistically significant improvements in all planes, as shown in Table 3.

Among the radiological measurements, talonavicular coverage angle on the left and right foot was correlated with an improved quality of life (left: Spearman r = 0.51, *p* = 0.0022; right: Spearman r = 0.38, *p* = 0.02).

All the patients had a follow-up period of a minimum of 2 years. None were lost in the follow-up period.

The statistical power of the study for a total of 69 patients divided into two groups (*n* = 33 in the study group, *n* = 36 in the control group) was 100%.

## 4. Discussion

In order to treat the flexible flatfoot, we have followed the clinical aspects of a fallen longitudinal arch and pain, as well as the radiological angles (calcaneal pitch, Meary, Kite face and profile and talonavicular coverage angles).

The goal of our study was to track the pediatric patients with flexible flatfoot deformity whose symptoms were interfering with their daily activities, but, more importantly, they significantly affected their sport performance. After recognizing those patients, the challenge for both surgeons and patients was to test their sport skills and check for improvements.

In introduction, we have selected one of the novel therapies (subtalar arthroereisis) in order to raise the quality of life and sport activities of our patients. This technique is meant to align the subtalar joint properly in pediatric patients, and involves the insertion of an implant which can be made of metal or synthetic material [39]. In our case, the implants used for the surgery were titanium made. Being a minimally invasive procedure, the patients were allowed to walk while wearing the cast and, after it was removed, they were advised to resume their normal gait. Initially, conservative treatments such as insoles, orthopedic shoes or physiotherapy may be used, but they did not prove very effective according to many studies [40,41,42]. Hsieh RL et al. debated the efficiency of customized insoles in terms of comfort, physical health and function, but with no progress for walking speed, global function or psychosocial health [42]. However, we recommended physiotherapy to those patients who required more time to recover their normal gait postoperatively.

No previous study verified postoperative recovery regarding sport activity, except for Martinelli et al. who observed that 91.8% of patients returned to sport [43]. With it being difficult to check if the patients truly returned to sport activities, our novel study evaluated the physical improvements by challenging the children at a 100 m sprint run. We tested the sprint run for the same child before the surgery and 6 months after in order to compare each child’s results. Another control group of healthy children was tested for the sprint run so as to have a normal comparison group. As seen from our results, compared to initial preoperative sprint runs, the postoperative values were much closer to the normal healthy group.

Body mass index (BMI) is a parameter often taken into consideration throughout studies while investigating the occurrence and severity of flexible flatfoot [3,44,45,46,47]. Thus, we measured our patients in order to minimize errors during physical testing which could have been related to weight gain or loss. BMI had a normal value for every individual from the study group, and, so as to reduce the risk of bias, it was calculated before sustaining the physical trial, preoperatively, and 6 months postoperatively as well.

After performing the surgery, all AOFAS scores were improved, with these results being similar to Megremis’ study [48]. Regarding the correction of the angles, their values were improved and associated with a higher quality of life, excepting calcaneal pitch.

Fernández de Retana et al. used the age range of 7–14 to evaluate the role of subtalar arthroereisis in flatfoot reconstruction, while the youngest patients in our study were operated on at the age of 9 [49]. A younger age group would have been irrelevant for surgical treatment considering that almost all children have flexible flatfeet until the age of five [5].

Mosca affirmed that subtalar arthroereisis may not have a clear indication and, in addition, the complication (misalignment, impingement pain, osteonecrosis, calcaneus fracture) rate varies between 3.5–11% according to recent studies [1,6,50,51,52,53,54]. In our study, surgery indication was based on clinical examination and radiological assignment; no side effect was reported and the quality of life increased after the surgery. 

Several limitations can be observed in our study, among which we can list the small number of only 33 patients, or the follow-up time of only 2 years. It should also be noted that the patients in our study group were patients with flexible flatfeet, with a moderate form of Achilles tendon shortening for which arthroereisis surgery was very suitable.

The strengths in our study were the homogeneity of the surgical technique, which was identical for all patients, and also the homogeneity between the study and control groups in terms of age, sex and BMI. Compared to previous publications, it is the first one to study and evaluate the physical improvements after arthroereisis in a flexible flatfoot cohort and compare results to normal healthy children.

Future studies should follow and compare, both in terms of quality of life, but also in terms of sports performance, both flexible flatfeet with Achilles tendon shortening, but also more severe flatfoot shapes for which interventions such as lateral column lengthening (Evans’ osteotomy modified by Mosca) or extra-articular subtalar arthrodesis (Grice) are indicated.

## 5. Conclusions

Flatfoot in children and adolescents may be a condition in which the quality of life and sports performance are decreased, compared to healthy children.

Arthroereisis is a minimally invasive surgical procedure with a short recovery time and a short period of resumption of sports activities, which can be useful in certain types of flexible flatfeet. Our study proved that it has a positive impact on our patients, curing symptoms such as painful gait and fatigue or improving the aesthetical aspect on all study subjects.

A long-term follow-up is needed so as to observe the effects of the subtalar implant over time.

## Figures and Tables

**Table 1:** Study and control groups data including preoperative and postoperative age, body mass index and quality of life score. M = male, F = female, BMI = body mass index, QoL = quality of life, S.D. = standard deviation, CI = confidence interval.

	Study Group				Control Group		
Total:	33 Patients (19 M, 14 F)				36 Children (20 M, 16 F)		
		Age (years)	BMI	QoL	Age (years)	BMI	QoL
Preoperative values:	Average	11.93	19.1	37.39	11.72	17.01	98.02
	S.D.	1.56	3.15	14.2	1.89	1.84	0.5
	95% CI	11.93 ± 0.55	19.1 ± 1.11	37.39 ± 5.03	11.72 ± 0.64	17.01 ± 0.62	98.02 ± 1.02
Postoperative values:	Average	12.43	19.44	98.84			
	S.D.	1.56	2.39	2.29			
	95% CI	12.43 ± 0.55	19.44 ± 0.85	98.84 ± 0.81			
*p*		0.54	<0.0001	<0.0001			

**Table 2:** Running time data between the study group and control group. Study Group was separated between preoperative and postoperative data. s = seconds, S.D. = standard deviation.

	Study Group		Control Group
	Preoperative Data	Postoperative Data	
Number of patients	33	33	36
Mean time (s)	24.55	22.30	20.94
S.D.	1.95	1.87	3.20
Max. time (s)	29.02	25.18	23.61
Min. time (s)	19.00	18.30	14.96
CI 95%	[23.7, 24.3]	[21.7, 22.3]	[19, 21]
*p*	<0.0001		0.01

**Table 3:** Radiological measurements in flatfoot, before and after arthroereisis surgery.

Preoperative					Postoperative					Wilcoxon Test
	Min.	Max.	Median	CI 95%		Min.	Max.	Median	CI 95%	*p* Value
Pitch (left)	10°	30°	15°	[16.52 ± 1.45]	Pitch (left)	12°	30°	19°	[19.18 ± 1.35]	<0.0001
Pitch (right)	9°	28°	15°	[16.67 ± 1.59]	Pitch (right)	12°	28°	19°	[19.42 ± 1.45]	<0.0001
Meary (left)	2°	24°	10°	[10.76 ± 1.63]	Meary (left)	−1°	9°	1°	[2 ± 0.71]	<0.0001
Meary (right)	2°	20°	11°	[10.42 ± 1.7]	Meary (right)	0°	6°	2°	[2.06 ± 0.549]	<0.0001
Kite face (left)	19°	61°	36°	[37.3 ± 3.41]	Kite face (left)	17°	39°	25°	[27 ± 2.18]	<0.0001
Kite face (right)	21°	52°	35°	[35.94 ± 3.15]	Kite face (right)	14°	40°	27°	[27.7 ± 2.27]	<0.0001
Kite profile (left)	31°	66°	56°	[53.76 ± 2.79]	Kite profile (left)	26°	58°	49°	[47.15 ± 2.58]	<0.0001
Kite profile (right)	34°	66°	55°	[52.94 ± 2.33]	Kite profile (right)	27°	56°	48°	[46.64 ± 2.39]	<0.0001
Talonavicular coverage angle (left)	5°	35°	21°	[20.36 ± 3.08]	Talonavicular coverage angle (left)	1°	16°	6°	[6.15 ± 1.16]	<0.0001
Talonavicular coverage angle (right)	5°	39°	19°	[19.36 ± 3.11]	Talonavicular coverage angle (right)	0°	15°	5°	[5.36 ± 1.12]	<0.0001

## Data Availability

All data are registered at “Grigore Alexandrescu” Children’s Emergency Hospital, Bucharest, Romania.
